# Self-supervised sparse coding scheme for image classification based on low rank representation

**DOI:** 10.1371/journal.pone.0199141

**Published:** 2018-06-20

**Authors:** Ao Li, Deyun Chen, Zhiqiang Wu, Guanglu Sun, Kezheng Lin

**Affiliations:** 1 Postdoctoral Station of School of Computer Science and Technology, Harbin University of Science and Technology, Harbin, China; 2 Department of Electrical Engineering, Wright State University, Dayton, OH, United States of America; 3 Department of Electronic and Information Engineering, University of Tibet, Lhasa, China; University of Virginia, UNITED STATES

## Abstract

Recently, sparse representation, which relies on the underlying assumption that samples can be sparsely represented by their labeled neighbors, has been applied with great success to image classification problems. Through sparse representation-based classification (SRC), the label can be assigned with minimum residual between the sample and its synthetic version with class-specific coding, which means that the coding scheme is the most significant factor for classification accuracy. However, conventional SRC-based coding schemes ignore dependency among the samples, which leads to an undesired result that similar samples may be coded into different categories due to quantization sensitivity. To address this problem, in this paper, a novel approach based on self-supervised sparse representation is proposed for image classification. In the proposed approach, the manifold structure of samples is firstly exploited with low rank representation. Next, the low-rank representation matrix is used to characterize the similarity of samples in order to establish a self-supervised sparse coding model, which aims to preserve the local structure of codings for similar samples. Finally, a numerical algorithm utilizing the alternating direction method of multipliers (ADMM) is developed to obtain the approximate solution. Experiments on several publicly available datasets validate the effectiveness and efficiency of our proposed approach compared with existing state-of-the-art methods.

## Introduction

Sparse representation has attracted great interest recently due to its powerful ability to model images, where it is assumed that an image can be represented by a linear combination of a few atoms of a basis set called a dictionary. It has achieved impressive performance on many computer vision tasks, such as image restoration, compressive sensing, tracking and classification [1–5]. In this paper, we focus on the sparse image classification problem. Many studies have been performed on sparse-based image classification in recent years. J. Wright [6] et al originally proposed the general sparse representation-based classification framework for face recognition. In their method, the sparse representation of a test sample can be computed by taking the training samples as a dictionary, and recognition is viewed as classifying among the multiple linear regression models by the representation errors. The promising results show the robustness of their method to occlusion and disguise. L. Zhang et al pointed out that the good performance of SRC profited not only from sparsity but from the collaboration among samples as well, and proposed the collaborative representation classification (CRC) method to obtain more accurate recognition results [7]. Moreover, they also suggested that sufficient samples were another essential factor affecting the recognition rate in an SRC-based framework. To reveal the intrinsic classification mechanism, a probabilistic collaborative representation method (ProCR) was proposed by Cai et al [8], in which the probability that a test sample belongs to the collaborative subspace of all classes was defined. Consequently, a ProCR-based classifier was designed to achieve excellent performance. Based on the idea of bag-of-features, [9] proposed an effective locality-constraint linear coding (LLC) scheme. Unlike the SRC, LLC projected the descriptor into its local-coordinate systems and the max pooling was performed on the projected codes to generate the final representation. To mitigate the performance degradation on datasets consisting of images with various camera orientations, a discriminative sparse coding approach was proposed to extract an affine-invariant feature and a classifier using AdaBoost was developed by taking affine sparse codes as the input. Fang et al [10] proposed a model to learn a non-negative sparse graph (NNSG), by which the classification is realized with an iterative supervised learning model to propagate the label information. To further improve representation ability, several extensive works on dictionary learning are studied for pattern classification. In [11], the discriminative KSVD (DKSVD) algorithm was first presented to unify the dictionary and classifier learning into the same framework. Next, a label consistent KSVD (LCKSVD) method was proposed by Z. L. Jiang to realize more discriminative sparse coding [12]. In LCKSVD, the label information of atoms was considered to enforce discriminability during dictionary learning. Similar to the DKSVD, classifier learning was also combined with the reconstruction error of dictionary learning to form a unified objective function. In [13], fisher discriminative dictionary learning was proposed for image classification in which, with the fisher criterion, not only was the representation residual used to distinguish the difference among classes, but also both of the scatters within-class and between-class are optimal. However, computational complexity and insufficient samples are two main drawbacks of the aforementioned dictionary learning algorithms. From another view, classification can be seen as a task to separate the samples lie in different linear subspaces. Therefore, the model that can capture the subspace structure among the samples is believed to be very helpful in pattern classification. Recently, the low rank representation (LRR) has attracted more and more interest with applications to image classification tasks [14]. It is noted that the LRR exhibits a remarkable ability in exploring the global manifold structure of data, which is a useful technique to analyze data drawn from multiple subspaces. C. Chen [15] proposed a low rank-based decomposition model with structural incoherence. It enforced the incoherence among the low rank matrix of different classes with an extra regularization, which helps to remove the noise from the contaminated data and provides additional discrimination for classification. Zhang et al [16] established a model with joint LRR and sparse representation, in which both the sparsity and low rank spatial consistency are exploited simultaneously. The preserved local structural information in the coding vector is more helpful for classification. Next, a structured LRR (SLRR) was further learned in a supervised way by [17]. In [17], an ideal LRR matrix was constructed to guide dictionary learning. Then, a simple linear classifier on the low rank representation matrix under the learned dictionary can also obtain promising results. Based on the viewpoint of supervised learning, a low rank and sparse representation (LRSR) model was studied by Zhuang et al [18], where the global mixture of subspace and local liner structure were both captured to construct a non-negative graph embedded in the semi-supervised classification.

Based on the above observations, here we propose a novel self-supervised sparse coding scheme for image classification. The main drawback of the conventional SRC-based methods is that it codes the samples independently and the mutual dependence among samples is consequently ignored. In our method, considering the advantages of LRR and sparsity in characterizing the manifold structure and local feature respectively, the test dataset is represented with joint sparse and low rank constraints to explore the consistency of similar samples. Next, the representation matrix is used to construct an effective regularization constraint to extend the SRC-based framework and provide maximal preservation of the similarity of features of similar samples. Furthermore, a numerical algorithm based on the alternating direction method of multipliers (ADMM) is developed to solve the novel objective function in our proposed sparse coding scheme.

The remainder of this paper is organized as follows. The overall framework of the proposed method is detailed in the next Section. The ‘Numerical algorithm’ section presents the numerical scheme designed for solving the novel objective function. Experimental results on several publicly available datasets and analysis are reported in ‘Experiments and discussion’ section to validate the effectiveness of the proposed scheme. Conclusions are given in the final section.

## Self-supervised sparse coding scheme

### Self-supervised matrix construction

Given *Y* = [*Y*_1_,*Y*_2_,*Y*_3_,⋯] ∈ *R*^*d*×*N*^ (*Y*_*i*_ ∈ *R*^*d*^), whose columns are the test samples drawn from independent subspaces (different classes), each sample of *Y* can be represented with the LRR model by a linear combination of atoms in dictionary *D* ∈ *R*^*d*×*n*^ as follows

$$\underset{Z}{\min}\left(rank(Z)\right)\;,\;s.t.\;Y=DZ$$ (1)

where *Z* ∈ *R*^*n*×*N*^ and *rank*(*Z*) denote the coefficient matrix and its rank. The purpose of the above objective function is to seek a representation matrix to capture the low-dimensional intrinsic structure of the dataset *Y*. However, the minimization in (1) cannot be solved directly. Generally, *rank*(*Z*) is replaced by its relaxed version as the following formulation.

$$\underset{Z}{\min}{\left\|Z\right\|}_{*}\;,\;s.t.\;Y=DZ$$ (2)

where ‖•‖_*_ denotes the nuclear norm as ${\left\|Z\right\|}_{*}=\sum_{i}\sigma_{i}\left(Z\right)$, the sum of the singular values of the matrix *Z*.

Nevertheless, in real applications, the datasets are often inevitably contaminated. Hence, to make the model more robust, an extra noise term could be introduced into the model in (2) as

$$\begin{array}{l} \underset{Z,E}{\min}\left\{{\left\|Z\right\|}_{*}+\lambda {\left\|E\right\|}_{1}\right\}\\ \;s.t.\;Y=DZ+E \end{array}$$ (3)

where *E* ∈ *R*^*d*×*N*^ denotes the noise term, and the *l*_1_-norm is forced on it with the sparsity assumption, and *λ* is a scalar to balance the two terms in the objective function.

Specially, when *D* is identity matrix, the model in Eq (3) is the convex relaxation version of the so-called robust principal component analysis(RPCA) model in [19], which aims to recover the low-rank and sparse components embedded in the observation matrix *Y* respectively. To further improve the performance of numerical solution, Gu et al[20] proposed a pursuit algorithm based on alternating minimization to solve the model as follows.

$$\begin{array}{c} \underset{U,V,S}{\arg\;\min}\left\{\frac{1}{2}{\left\|Y-D\left(UV^{T}+S\right)\right\|}_{F}^{2}\right\}\\ s.t.\;{\left\|S\right\|}_{0}\leq s \end{array}$$ (4)

where *UV*^*T*^ (*U* ∈ *R*^*d*×*r*^, *V* ∈ *R*^*N*×*r*^) denotes a automatically satisfied low-rank component, and *S* ∈ *R*^*d*×*N*^ denotes the sparse error matrix. *s* is a tuning parameter to constrain the sparsity degree.

As pointed out by [21], a low-rank constraint is good for seeking a representation matrix, which reveals the relationship between each sample and bases in dictionary from the global manifold structure. However, the locality structure is ignored, which generates the imperfection on the feature selection from the perspective of independent samples. To address this issue, a joint low-rankness and sparse representation model was established as follows

$$\begin{array}{c} \underset{Z,E}{\min}\left\{{\left\|Z\right\|}_{*}+\gamma {\left\|Z\right\|}_{1}+\lambda {\left\|E\right\|}_{1}\right\}\\ s.t.\;Y=DZ+E \end{array}$$ (5)

where the additive second term is used to constrain the sparsity on the representation coefficient matrix, by which the model in Eq (5) can preserve the global manifold structure and locality structure simultaneously in a unified framework. Furthermore, replacing *D* with dataset *Y* itself, it is believed that the representation matrix can better reveal the relationship between the samples from both global and local perspective.

In fact, RPCA and the model in Eq (4) are the methods for component separation. It is worth noting that the solutions of the these two models are two irrelevant components that represent different structures embedded in the observation matrix. They are constrained with low-rank and sparsity respectively, which is a popular method in the low-rank recovery problems. Nevertheless, different from them, for the classification task in this paper, the model in Eq (5) aims to constrain both of the low-rank and sparsity on the same matrix *Z* but not two separate superposition components, which will make the coefficient matrix *Z* not only explore the global relevant structure but also select the local sparsity structure. That is, the coefficient matrix *Z* can meet the two constraints simultaneously, which is believed to help to present the correlation of samples more effectively. In other words, only with the low-rank constraint, *Z* can capture the global latent subspace structures but cannot guarantee the sufficient local sparsity of its column vectors. Hence, the extra sparsity constraint enforces *Z* to obtain higher coefficients with respect to samples from the same subspace but lower ones with respect to the samples from other irrelevant subspaces.

With the recently developed linearized alternating direction method [22], the optimization problem in Eq (5) can be solved iteratively. An auxiliary variable is introduced to make the objective function separable as the following form

$$\begin{array}{l} \underset{Z,P,E}{\min}\left\{{\left\|Z\right\|}_{*}+\gamma {\left\|P\right\|}_{1}+\lambda {\left\|E\right\|}_{1}\right\}\\ \;s.t.\;Y=YZ+E,P=Z \end{array}$$ (6)

The augmented Lagrangian function of Eq (6) is defined as

$$\begin{array}{l} L_{\mu }\left(Z,P,E,M_{1},M_{2}\right)={\left\|Z\right\|}_{*}+\gamma {\left\|P\right\|}_{1}+\lambda {\left\|E\right\|}_{1}+\langle M_{1},Y-YZ-E\rangle \\ \;+\langle M_{2},P-Z\rangle +\frac{\mu }{2}\left({\left\|Y-YZ-E\right\|}_{F}^{2}+{\left\|P-Z\right\|}_{F}^{2}\right) \end{array}$$ (7)

where ⟨*A*,*B*⟩ = *tr*(*A*^*T*^*B*), *tr*(•) denotes the sum of diagonal elements, and *M*_1_ and *M*_2_ are the Lagrangian multipliers.

Eq (7) can be rewritten as

$$\begin{array}{c} L_{\mu }\left(Z,P,E,M_{1},M_{2}\right)={\left\|Z\right\|}_{*}+\gamma {\left\|P\right\|}_{1}+\lambda {\left\|E\right\|}_{1}+H_{\mu }\left(Z,P,E,M_{1},M_{2}\right)\\ -\frac{1}{2\mu }\left({\left\|M_{1}\right\|}_{F}^{2}+{\left\|M_{2}\right\|}_{F}^{2}\right) \end{array}$$ (8)

where $H_{\mu }\left(Z,P,E,M_{1},M_{2}\right)=\frac{\mu }{2}\left({\left\|Y-YZ-E-M_{1}/\mu \right\|}_{F}^{2}+{\left\|P-Z-M_{2}/\mu \right\|}_{F}^{2}\right)$.

With some algebra, the update scheme based on the gradient term of *H*_*μ*_(•) at the *k*-th iteration can be shown as follows

$$Z^{k+1}=\arg\;\min\left\{\frac{\beta \mu_{k}}{2}{\left\|Z-Z^{k}+\left(\begin{array}{l} -Y^{T}\left(Y-YZ^{k}-E^{k}-M_{1}^{k}/\mu_{k}\right)\\ +\left(P^{k}-Z^{k}-M_{2}^{k}/\mu_{k}\right) \end{array}\right)/\beta \right\|}_{F}^{2}+{\left\|Z\right\|}_{*}\right\}$$ (9)

$$P^{k+1}=\arg\;\min\left\{\frac{1}{2}{\left\|P-Z^{k+1}-M_{2}^{k}/\mu \right\|}_{F}^{2}+\frac{\gamma }{\mu_{k}}{\left\|P\right\|}_{1}\right\}$$ (10)

$$E^{k+1}=\arg\;\min\left\{\frac{1}{2}{\left\|E-\left(Y-YZ^{k+1}-M_{1}^{k}/\mu \right)\right\|}_{F}^{2}+\frac{\lambda }{\mu_{k}}{\left\|E\right\|}_{1}\right\}$$ (11)

Assuming that,

$$Z^{k}-\left(\begin{array}{l} -Y^{T}\left(Y-YZ^{k}-E^{k}-M_{1}^{k}/\mu_{k}\right)\\ +\left(P^{k}-Z^{k}-M_{2}^{k}/\mu_{k}\right) \end{array}\right)/\beta =U\Lambda V^{T}$$ (12)

The nuclear norm minimization problem in Eq (9) can be solved by

$$Z^{k+1}=US_{\tau }\left(\Lambda \right)V^{T}$$ (13)

where *S*_*τ*_(Λ) = sgn(Λ)max(|Λ|−τ,0), and *τ* is the shrinkage parameter as $\tau =\frac{1}{\beta \mu_{k}}$.

The two subproblems in Eqs (10) and (11) are the classical *l*_1_ norm minimization, which can be easily solved with the soft shrinkage operator (SSO) *S*_*τ*_(•) on their observation matrix respectively.

As for the Lagrangian multipliers, the following update formulation can be used:

$$M_{1}^{k+1}=M_{1}^{k}+\mu_{k}\left(Y-YZ^{k+1}-E^{k+1}\right)$$ (14)

$$M_{2}^{k+1}=M_{2}^{k}+\mu_{k}\left(P^{k+1}-Z^{k+1}\right)$$ (15)

The detailed scheme for solving problem in Eq (6) is outlined in Table 1 as follows.

**Table 1:** Algorithm for solving objective function in Eq (6).

Algorithm 1
Initial: Dataset *Y*, $Z^{0}=P^{0}=E^{0}=M_{1}^{0}=M_{2}^{0}=0$; **while** *k* < *K* 1. Compute *Z*^*k*+1^ by solving subproblem (9) with Eq (13); 2. Compute *P*^*k*+1^ by solving subproblem (10) with SSO; 3. Compute *E*^*k*+1^ by solving subproblem (11) with SSO; 4. Update the Lagrangian multipliers with Eqs (14) and (15); 5. *k* ← *k* + 1; **end while**

With Algorithm 1, the low-rank and sparse representation coefficient matrix *Z* is obtained, which can better simultaneously preserve both the global manifold and locality structure of test dataset *Y*. Considering the symmetric property of sample consistency and the non-negative requirement on the regularizer, a self-supervised matrix can be constructed as follows:

$$W=\frac{\left|Z\right|+\left|Z^{T}\right|}{2}$$ (16)

where |•| denotes the element-wise absolute value operation. Due to the revealed intrinsic structure of matrix *Z*, *W* can be utilized as an effective self-supervised mechanism on the consistency of similar samples, which is helpful to enforce the mutual dependency among samples to further improve the classification accuracy. The details will be discussed in the next subsection.

### Detailed coding scheme

Given the training dataset *X* = [*X*_1_,*X*_2_,⋯*X*_*i*_,⋯], the conventional SRC-based model is presented as

$$\underset{A}{\min}\left\{\frac{1}{2}{\left\|Y-XA\right\|}_{F}^{2}+\eta {\left\|A\right\|}_{1}\right\}$$ (17)

where *X*_*k*_ denotes the subset samples in the *k*-th class, and *A* represents the coding coefficient matrix. Then, the following reconstruction residual error is used to classify the samples:

$$r\left(Y_{i}\right)=\underset{k}{\arg\;\min}\left\{{\left\|Y_{i}-X_{k}A_{i}^{k}\right\|}_{F}^{2}\right\}$$ (18)

where $A_{i}^{k}$ denotes the subset of *A*_*i*_ corresponding to class *k*, and *r*(*Y*_*i*_) denotes the final classification result about *Y*_*i*_.

From Eqs (17) and (18), it can be observed that the coding *A*_*i*_ of each sample has significant influence on the classification result. Naturally, a better coding scheme will improve the final accuracy. Nevertheless, the model in Eq (17) ignored the mutual dependency among the samples and coded each sample independently. It is noted that the model in Eq (17) may lead to an undesired fact that a similar sample may lie in different atoms of different classes, due to the sensitiveness of the sparse coding process. To address this problem, we propose a self-supervised coding scheme as follows:

$$\underset{A}{\min}\left\{\frac{1}{2}{\left\|Y-XA\right\|}_{F}^{2}+\eta {\left\|A\right\|}_{1}+\xi \sum_{i}\sum_{j}W_{ij}{\left\|XA_{i}-XA_{j}\right\|}_{2}^{2}\right\}$$ (19)

where *W*_*ij*_ presents the element in the (*i*,*j*) location of the self-supervised *W* obtained Eq (16), *η* and *ξ* are scalars to control the balance among the three terms. The first term in Eq (19) evaluates the reconstruction error, and the second term is the sparsity constraint on the coding matrix to preserve the locality structure of the sample. The purpose of the third term is to propagate the locality information of similar samples, which is used to preserve the consistency of features of similar samples. For each pair of samples (*Y*_*i*_,*Y*_*j*_), inspired by the excellent performance of *W* on capturing the intrinsic subspace structure of data, a larger *W*_*ij*_ means less distance between the two samples in the intrinsic subspace.

## Numerical algorithm

To handle the proposed model in Eq (19), we reformulate it as

$$\underset{A}{\min}\left\{\frac{1}{2}{\left\|Y-XA\right\|}_{F}^{2}+\eta {\left\|A\right\|}_{1}+\xi tr\left(XAS{\left(XA\right)}^{T}\right)\right\}$$ (20)

where *tr*(•) is the sum of diagonal elements of matrix, and *S* = *L*−*W*, *L* is a diagonal matrix with $L_{ii}=\sum_{j}W_{ij}$. Then, we introduce a slack variable to make the objective function separable as

$$\begin{array}{c} \underset{A,U}{\min}\left\{\frac{1}{2}{\left\|Y-XA\right\|}_{F}^{2}+\eta {\left\|A\right\|}_{1}+\xi tr\left(U^{T}SU\right)\right\}\\ s.t.\;XA=U^{T} \end{array}$$ (21)

Introducing the auxiliary matrix *H*, the Lagrangian function of Eq (21) is

$$\begin{array}{c} L_{\rho }\left(A,U,H\right)=\frac{1}{2}{\left\|Y-XA\right\|}_{F}^{2}+\eta {\left\|A\right\|}_{1}+\xi tr\left(U^{T}SU\right)+\langle H,XA-U^{T}\rangle +\frac{\rho }{2}{\left\|XA-U^{T}\right\|}_{F}^{2}\\ =\frac{1}{2}{\left\|Y-XA\right\|}_{F}^{2}+\eta {\left\|A\right\|}_{1}+\xi tr\left(U^{T}SU\right)+\frac{\rho }{2}{\left\|XA-U^{T}-H\right\|}_{F}^{2}-\frac{1}{2\rho }{\left\|H\right\|}_{F}^{2} \end{array}$$ (22)

Through ADMM, with some algebra, the variables can be updated as follows:

$$A^{k+1}=\arg\;\min\left\{\frac{1}{2}{\left\|Y-XA\right\|}_{F}^{2}+\eta {\left\|A\right\|}_{1}+\frac{\rho }{2}{\left\|XA-{\left(U^{k}\right)}^{T}-H^{k}\right\|}_{F}^{2}\right\}$$ (23)

$$U^{k+1}=\arg\;\min\left\{\frac{\rho }{2}{\left\|U-{\left(XA^{k+1}-H^{k}\right)}^{T}\right\|}_{F}^{2}+\xi tr\left(U^{T}SU\right)\right\}$$ (24)

$$H^{k+1}=H^{k}+\rho \left(XA^{k+1}-{\left(U^{k+1}\right)}^{T}\right)$$ (25)

With gradient descent, the optimization in (23) can be reformulated as

$$A^{k+1}=\arg\;\min\left\{\frac{\rho \zeta }{2}{\left\|A-A^{k}\right\|}_{F}^{2}+\langle \nabla Q_{A}\left(A^{k},U^{k},H^{k}\right),A-A^{k}\rangle +\eta {\left\|A\right\|}_{1}\right\}$$ (26)

where $Q\left(A,U^{k},H^{k}\right)=\frac{1}{2}{\left\|Y-XA\right\|}_{F}^{2}+\frac{\rho }{2}{\left\|XA-{\left(U^{k}\right)}^{T}-H^{k}\right\|}_{F}^{2}$. Further,

$$A^{k+1}=\arg\;\min\left\{\frac{\rho \zeta }{2}{\left\|A-A^{k}+\frac{1}{\rho \zeta }\nabla Q_{A}\left(A^{k},U^{k},H^{k}\right)\right\|}_{F}^{2}+\eta {\left\|A\right\|}_{1}\right\}$$ (27)

where ∇*Q*_*A*_(*A*^*k*^,*U*^*k*^,*H*^*k*^) = −*X*^*T*^ (*Y*−*XA*^*k*^)+*ρX*^*T*^ (*XA*^*k*^−(*U*^*k*^)^*T*^−*H*^*k*^). And then, the problem can be easily solved with the aforementioned SSO method.

Enforcing the first order derivative of formulation (21) to be zero, we can obtain

$$U^{k+1}=\rho {\left(\rho I+\zeta S\right)}^{-1}{\left(XA^{k+1}-H^{k}\right)}^{T}$$ (28)

With the solved coding matrix *A*, in our algorithm, a simple classification with reconstruction error is employed. Different from formulation (18), to be more adaptive to our proposed coding scheme, the reconstruction error is designed as follows:

$$r\left(Y_{i}\right)=\underset{k}{\arg\;\min}\left\{{\left\|XA_{i}-X_{k}A_{i}^{k}\right\|}_{F}^{2}\right\}$$ (29)

where sample *Y*_*i*_ is replaced by its reconstruction version. The overall algorithm is summarized in Table 2.

**Table 2:** Overall algorithm for proposed coding scheme.

Algorithm 2
Initial: test dataset *Y*, training dataset *X*, *A*^0^ = *U*^0^ = *H*^0^ = **0**; 1. Learning self-supervised matrix *W* by **Algorithm 1**. **While** *k* < *K* 2. Compute *A*^*k*^ by solving formulation (27); 3. Compute *U*^*k*^ with formulation (28); 4. Update *H*^*k*^ with formulation (25); 5. *k* ← *k* + 1; **end while**

## Experiments and discussion

**Fig 1:**
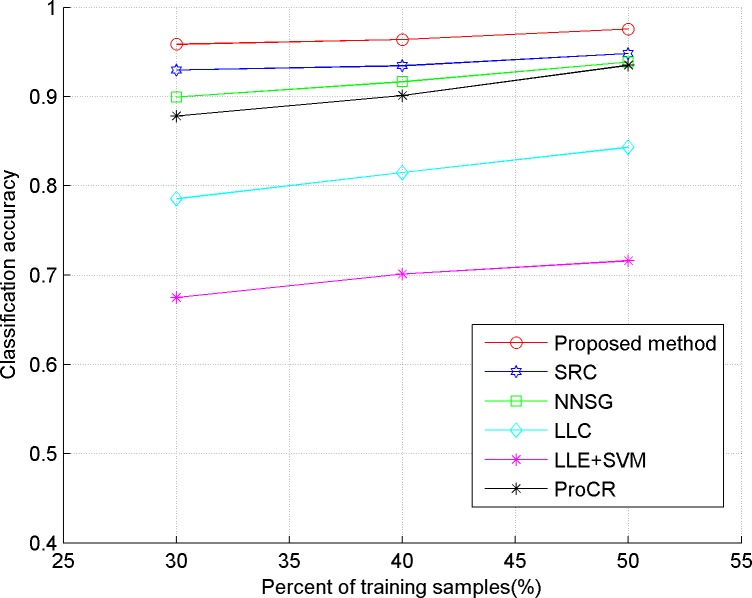
Classification accuracy of comparison methods on Extended YaleB.

**Fig 2:**
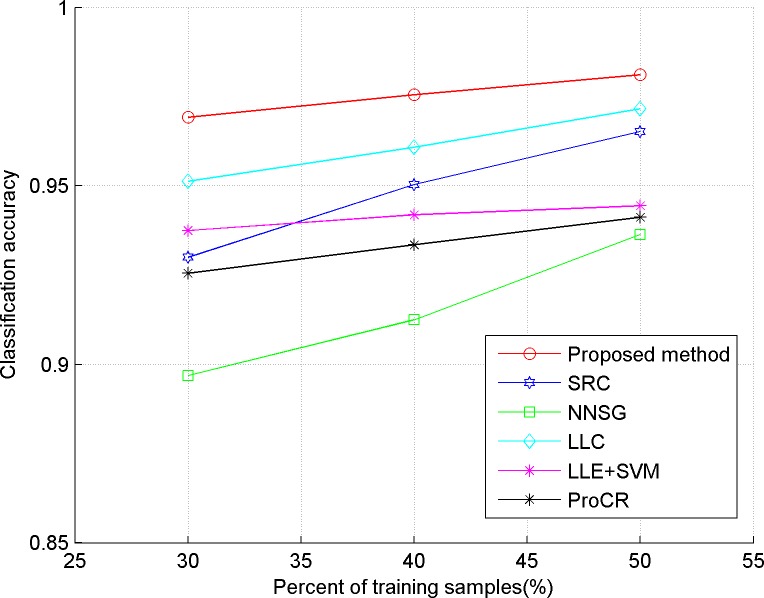
Classification accuracy of comparison methods on COIL20.

**Fig 3:**
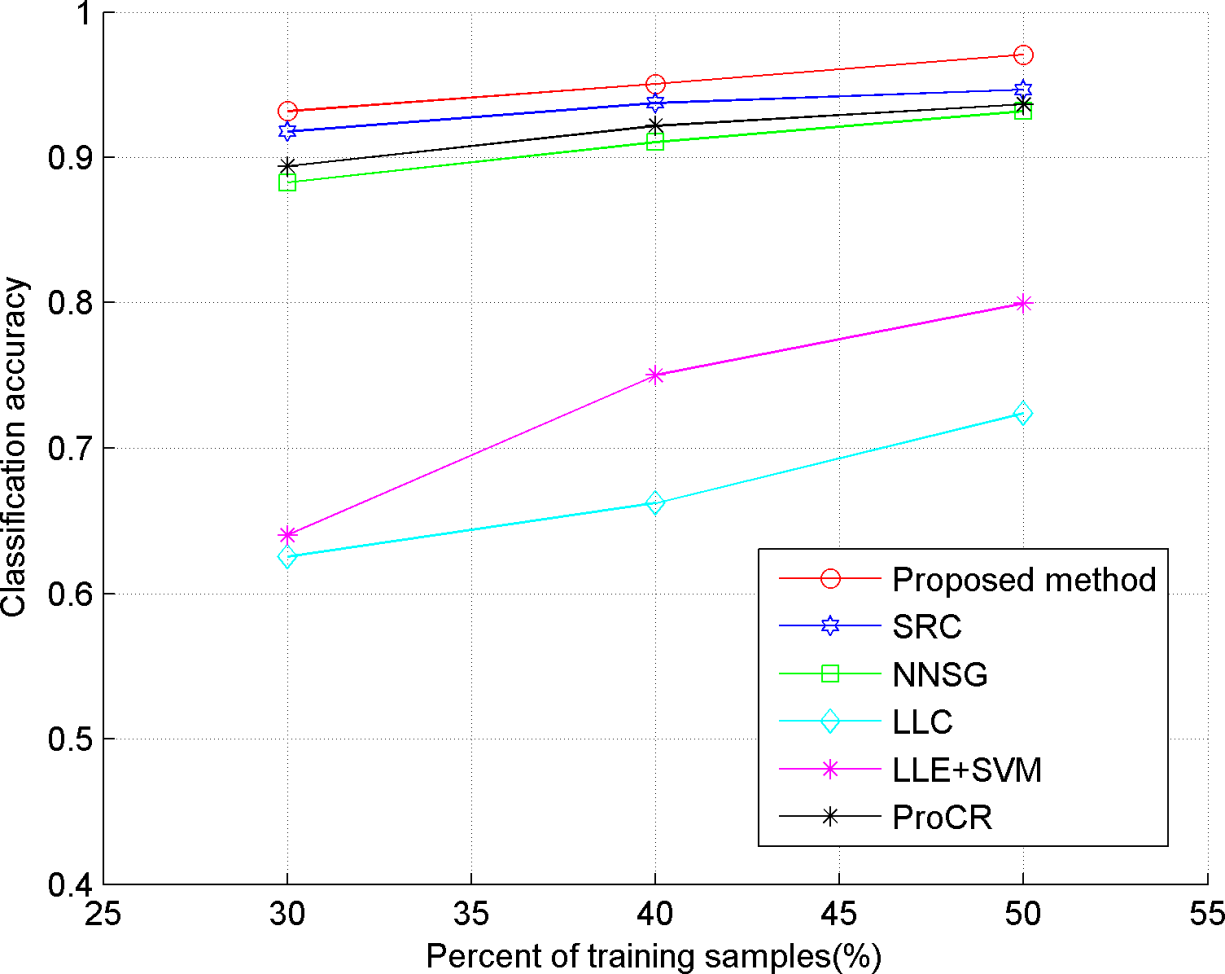
Classification accuracy of comparison methods on AR.

**Fig 4:**
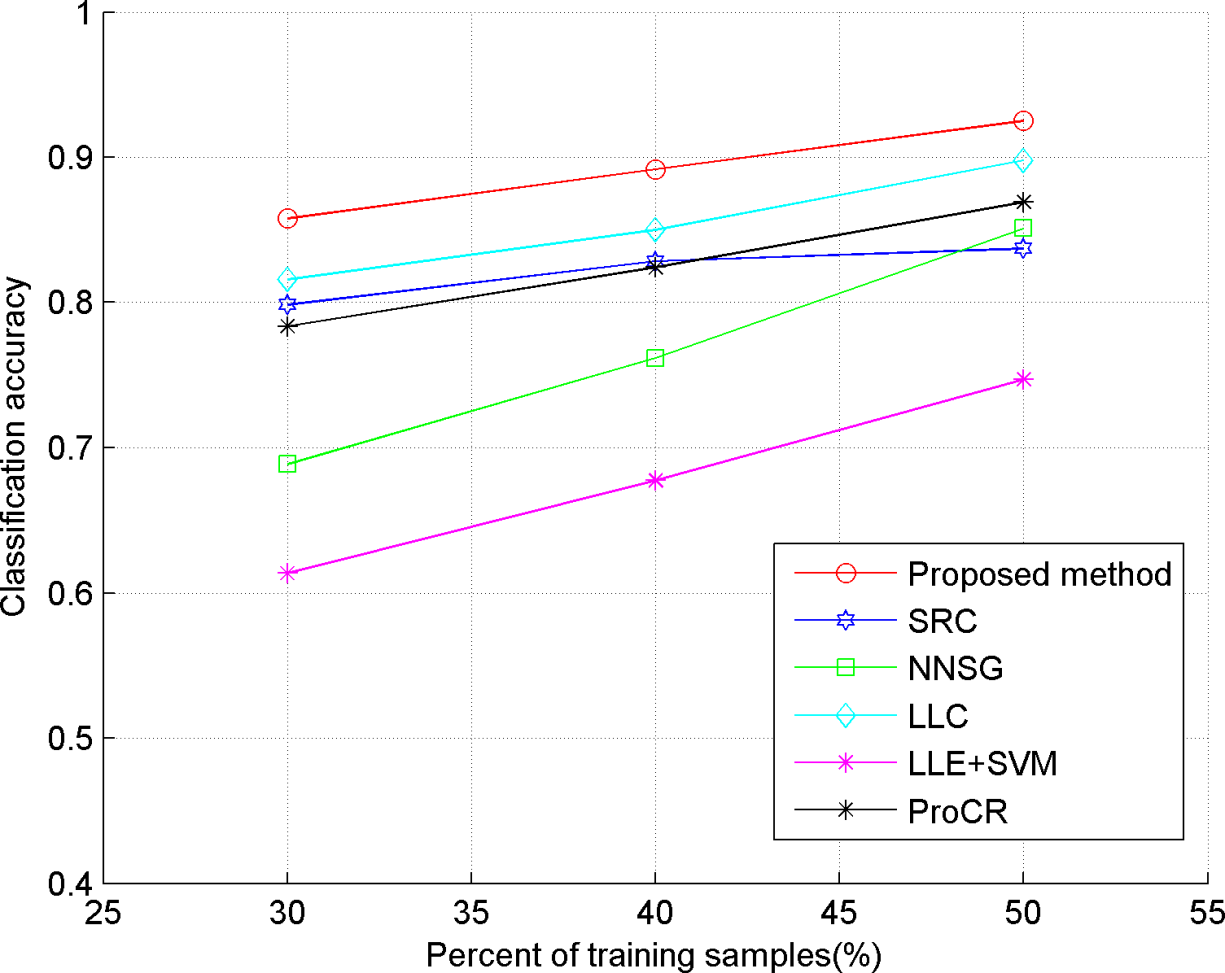
Classification accuracy of comparison methods on ORL.

**Fig 5:**
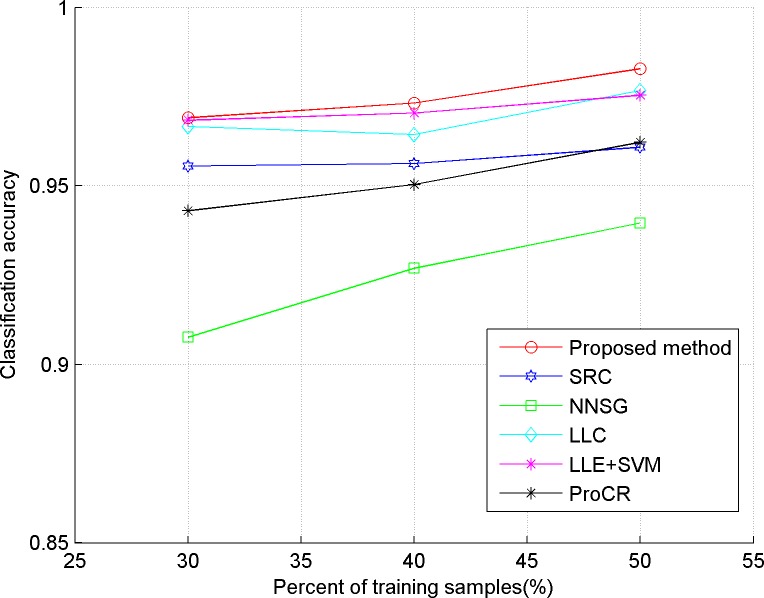
Classification accuracy of comparison methods on USPS.

In our experiments, we evaluate the proposed algorithm on five publicly available datasets, including three face datasets: Extended YaleB, AR, ORL, one object dataset: COIL-20, and a handwritten dataset: USPS. The comparison classification methods include SRC in [6], LLC in [9], LLE+SVM in [23], ProCR in [8], and NNSG in [10]. Considering the computational cost, two dimensional reduction methods are used in our experiment. "Eigenface", proposed in [24], is used for the face dataset, and PCA is implemented for the others. In our experiments, 30, 40 and 50 percent of the dataset are taken randomly as training samples respectively, and the remaining ones are used as test samples. Also, the test experiment is implemented five times for each method, and the average accuracy is reported as the final classification result, which is shown in Figs 1–5. The detailed description for dataset and setting is listed as follows.

### Datasets description and setting

#### Face dataset

The Extended YaleB face dataset includes 2414 frontal image of 38 persons, and each person has about 64 images with different lighting conditions. The ORL dataset contains 400 images of 40 persons, where 10 images with varying illumination, disguise, and facial expression, are provided for each person. As for the AR dataset, it consists of 3120 faces from 120 persons, and provides about 26 images for each person also with different lighting conditions, facial expression and occlusion (glasses or scarf). The samples of the Extended YaleB and ORL are cropped and resized to 32 × 32. The size of AR used in our experiments is 55 × 40. The dimension is reduced to 200.

#### Object dataset

The COIL20 contains 1440 images from 20 objects, and each object has 72 images obtained from continuous angles at intervals of five degree. In our experiments, the images are also cropped and resized to 32 × 32. The dimension is reduced to 100.

#### Handwritten dataset

The USPS dataset has 9298 handwritten digit images for ten digits from 0 to 9 and the size of each image is 16 × 16. In our experiments, the dimension of data is reduced to 100.

### Results analysis

From the results depicted in Figs 1–5, it can be seen that LLE+SVM, in which only few neighbors are used for structural embedding, achieves the worst results. Furthermore, all the comparison methods achieve a lower accuracy on ORL than on other datasets, which is due to the insufficient number of samples in each category of ORL. Therefore, it can be concluded that the collaborative dependency of samples is very important to preserve the structural information among the different categories. On the other hand, LLC takes the relationship between the sample and atom as the coding constraint, which weakens the collaboration of samples. So, LLC has poor classification results on face datasets (even worse than the conventional SRC), but shows some superior performances on the object category. By constructing a graph with non-negative sparse representation to control the labels consistency, NNSG shows competitive classification results. However, alternation between graph construction and supervised learning demands huge computation cost with the increasing number of samples. By exploiting the mutual dependency among the samples, the ProCR and our proposed method achieve better performance than others. Nevertheless, unlike ProCR, our proposed method aims to explore the relationship from both test samples and training samples. Moreover, better manifold structure and locality are explored by the sparse low-rankness model to propagate the self-supervised constraint mechanism between samples’ coding. As a direct result, our proposed coding scheme consistently achieves the best classification accuracy.

**Fig 6:**
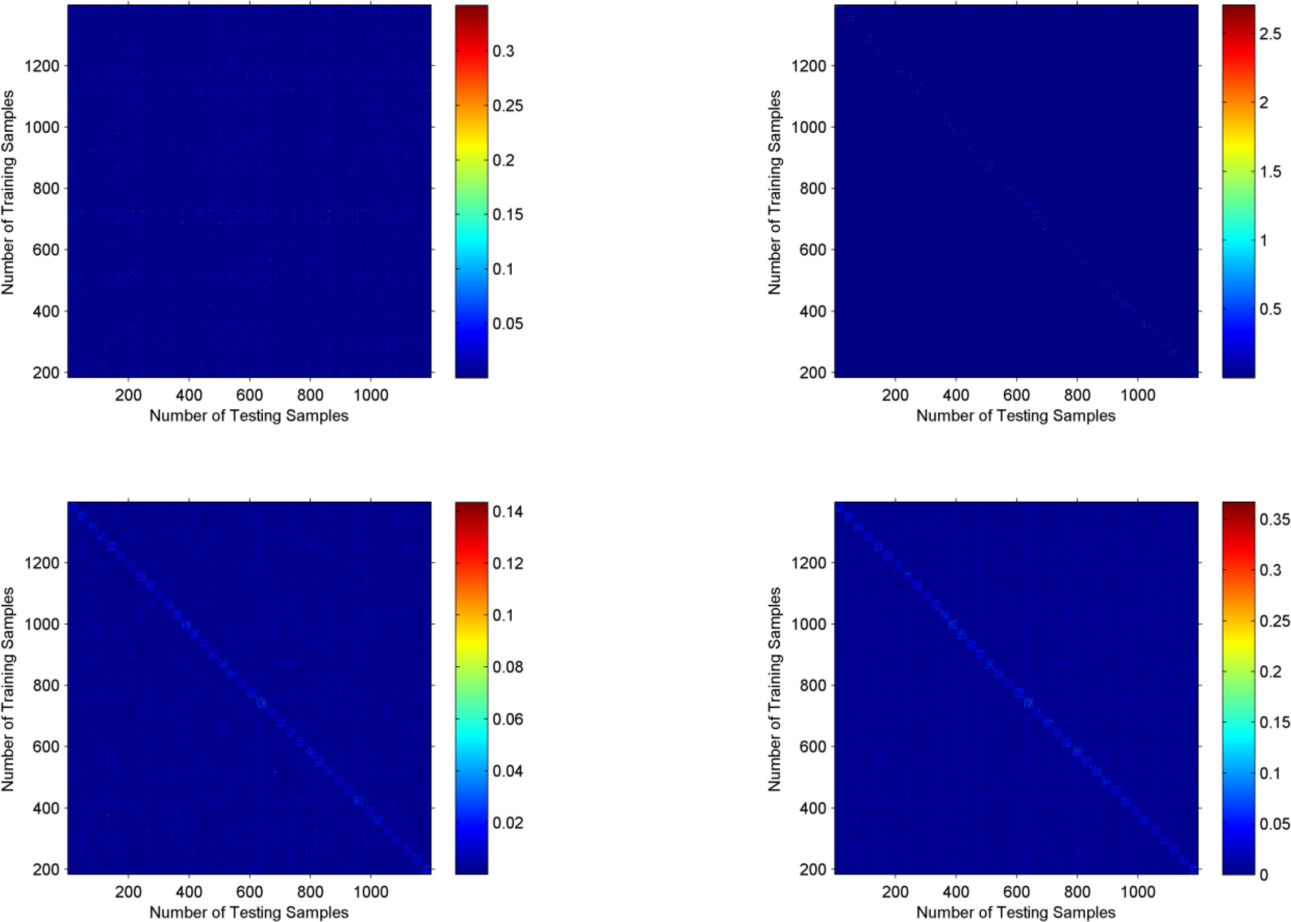
The coding matrices of Extended YaleB with different coding scheme. Top row: visulization of coding matrices of SRC(left) and LLC(right). Bottom row: visulization of coding matrices of ProCR(left) and our proposed method(right). The visualization results are obtained under the experiments with 50 percent of training samples.

To further verify the performance, the coding matrices of SRC, LLC, ProCR and the proposed scheme are shown in Fig 6. They are obtained under the experiments on Extended YableB with 50 percent training samples. Also, though the test samples are chosen randomly in the experiments, we reorder them here in terms of their category to present the structure more clearly. From Fig 6, it can be observed that the proposed coding scheme has obvious block-diagonal structure, which means that our method can better preserve the structural and locality information of similar samples in the same category to produce more discriminative results.

**Fig 7:**
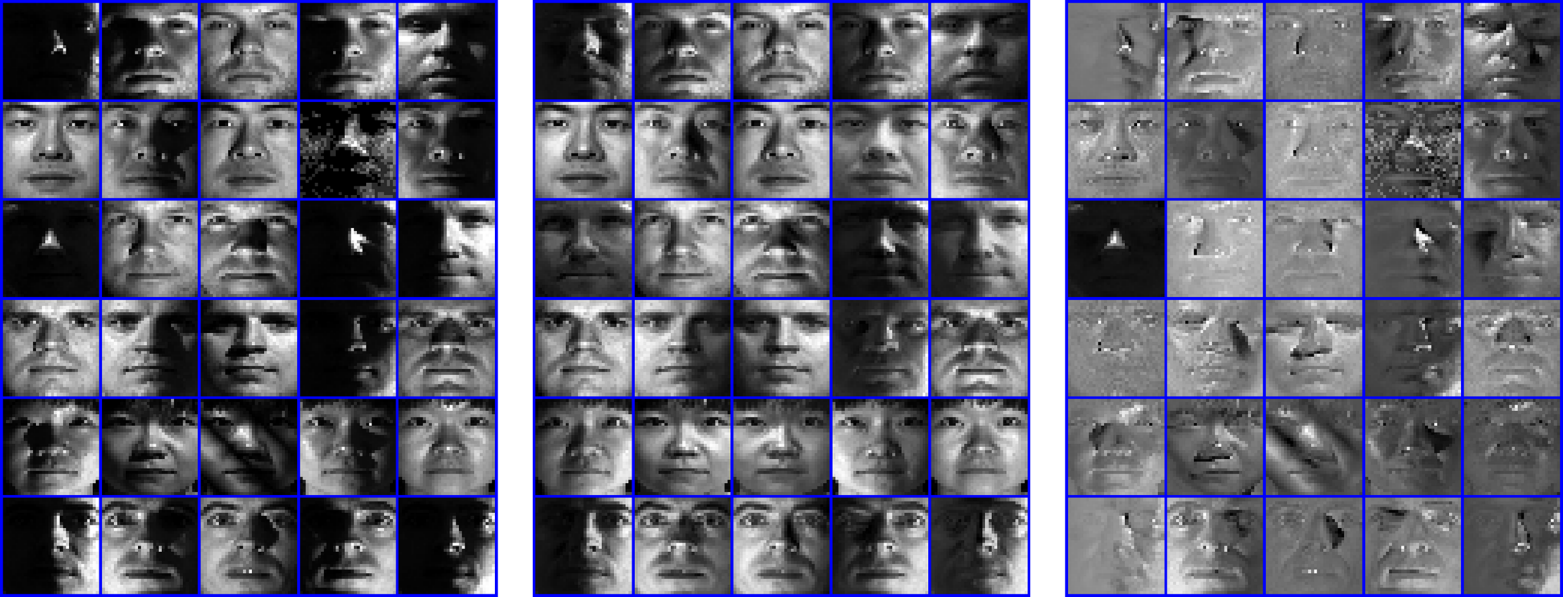
Visualization results on Extended YaleB. The original face samples are shown in the left, the synthesis face samples are shown in the middle and the residual errors are shown in the right.

For more clarity, we take Extended YaleB as example to show some ability of our proposed model versus illumination varying. To visualize the results, we don't reduce the dimensionality of sample and take the original image as training and test sample. Similarly, 32 images of each class are randomly selected as the training data *X* and remaining are used as *Y* for testing. With our proposed model, the self-supervised coding matrix *A* for test samples can be obtained. We randomly select 30 test images from the 6 classes(5 images for each class) as examples to show their synthesis results in Fig 7. For each test sample *y*_*i*_, its synthesis sample ${\hat{y}}_{i}^{s}$ can be obtained as ${\hat{y}}_{i}^{s}=XA_{i}$.

From Fig 7, it can be observed that the synthesis sample partly remove the illumination limitation. Actually, these promising results are obtained due to the collaborative representation among training samples. Furthermore, though the fidelity term ${\left\|Y-XA\right\|}_{F}^{2}$ in the proposed model enforces the synthesis sample close to the test sample, the residual component is still existed between them. Hence, some undesired components, such as illumination limitation in face, or appearance varying of object will be left in the residual errors.

**Fig 8:**
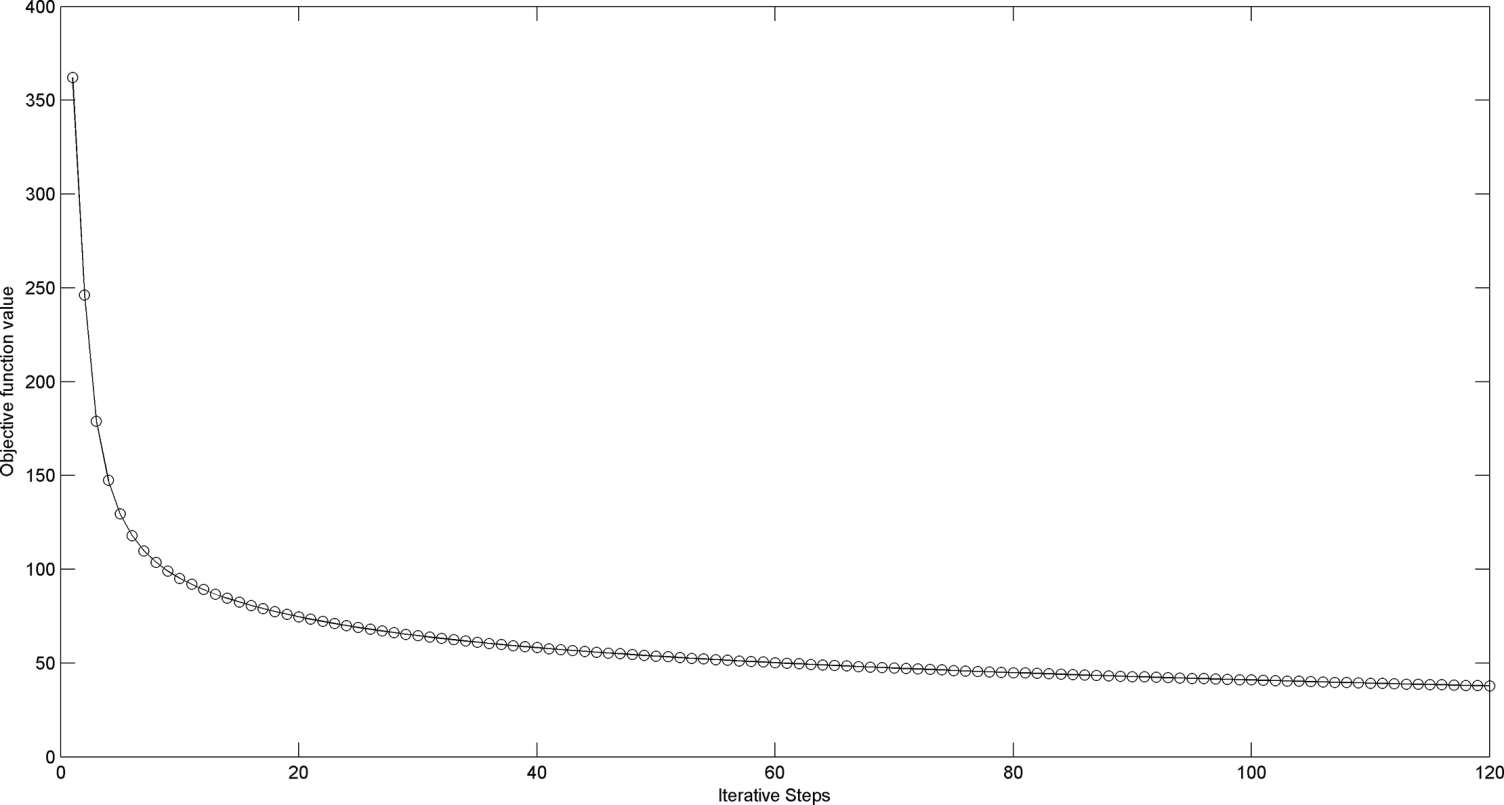
The convergence curve of Extended YaleB.

Finally, we will give some discussion on the computational complexity and convergence of our method. It can be observed that the computational cost of proposed model is mainly determined by Eqs (27) and (28). Without loss of generality, we assumes that the size of *X* and *A* are *d*_1_ × *n* and *n* × *n* respectively. In each iteration, the we firstly employ the SSO method whose complexity is O(*n*^2^). And then, a matrix pseudo inverse operation is applied for *U* whose complexity is O(*d*_1_*n*^2^). So, the total cost of our algorithm is O(*d*_1_*n*^2^ + *n*^2^), that is, the computational complexity is at most O(*d*_1_*n*^2^). For the convergence, we take the Extended YaleB as example to plot its objective function value versus the iterative steps in Fig 8. From Fig 8, we can see that the convergence curve of our proposed algorithm is decreased monotonically and enjoys the approximate inverse proportion convergence rate.

## Conclusions

To address the image classification problem, in this paper we propose a discriminative sparse coding approach, in which a representation matrix is first computed by joint low-rankness and sparse representation model to preserve the latent manifold structure and locality of test samples. Next, by incorporating the representation matrix, a self-supervised sparse coding model is established to improve the classification performance. Through this coding scheme, the mutual dependency between similar samples can be better explored and propagated, which will generate self-supervised mechanism to enforce the close coding for similar samples. Meanwhile, a more suitable reconstruction error is designed as the classification criterion. Moreover, we also provides an iterative numerical algorithm to solve the novel objective function in the proposed model based on ADMM. Several experiments on five public datasets clearly show that our proposed method outperforms existing state-of-the-art classification methods.
